# Immersive virtual reality for pediatric burn pain and early rehabilitation

**DOI:** 10.3389/fped.2026.1876793

**Published:** 2026-07-29

**Authors:** Liping Lu, Wenhui Li, Shuangshuang Ye, Ping Dai

**Affiliations:** 1 Department of Burns and Plastic Surgery, No. 910 Hospital of the Chinese People’s Liberation Army Joint Logistic Support Force, Quanzhou, China; 2 Health Management Center, Xiang’an Hospital of Xiamen University, Fujian, China

**Keywords:** burn rehabilitation, dressing change, immersive virtual reality, pediatric burns, procedural pain

## Abstract

Burn dressing changes are painful and distressing for children, and repeated procedural discomfort can reduce cooperation with wound care and early rehabilitation. This study was conducted in the burn unit of No.910 Hospital of the Chinese People's Liberation Army Joint Logistic Support Force between January 2023 and March 2026 to evaluate the effects of immersive virtual reality (VR) on procedural distress and treatment cooperation during repeated dressing changes and early rehabilitation in children with burns. Eighty pediatric patients requiring repeated dressing changes were randomly assigned to a control group or a VR group, with 40 children in each group. Children in the control group received routine burn care and standard distraction, whereas those in the VR group received routine care plus immersive VR animation during dressing changes and early rehabilitation sessions. Procedural pain was assessed using the Faces Pain Scale–Revised, procedure-related fear using the Children's Fear Scale, and behavioral distress using the COMFORT-B scale; treatment cooperation, rehabilitation completion, dressing duration, rescue analgesic use, and adverse events were also recorded. Baseline demographic and burn-related characteristics were similar between groups. No significant differences were found in pain, fear, or behavioral distress during the first dressing change, and a similar pattern was observed during the second dressing change. By the third dressing change, children in the VR group reported less pain (*p* = 0.0009), less fear (*p* = 0.0116), and lower behavioral distress (*p* = 0.0048) than those receiving routine care. The VR group also showed better dressing cooperation at the third dressing change (*p* = 0.0024) and better rehabilitation cooperation during the second and third rehabilitation sessions (*p* = 0.0352 and *p* = 0.0106, respectively). Exploratory sex-stratified analysis showed that VR-related improvements were reflected mainly in pain and fear among male patients and in pain and behavioral distress among female patients. Rehabilitation completion, dressing duration, and rescue analgesic use did not differ significantly between groups, and no serious adverse events occurred. These findings suggest that immersive VR may be a practical adjunct for making repeated pediatric burn care less distressing and more tolerable while improving cooperation during later treatment sessions.

## 1 Introduction

Burn injury is a leading cause of morbidity, hospitalization, and long-term disability in children (1). Even limited injuries often necessitate repeated medical visits, wound care, and rehabilitation, while families endure significant financial and emotional strain (2, 3). Pediatric burns differ from adult burns due to children's thinner skin, distinct body proportions, developmental vulnerability, and greater reliance on caregivers (4). Under identical thermal exposure, children are more likely than adults to sustain deeper burns, as less energy is required to damage thinner skin (5). Moreover, children are at higher risk for hypothermia, fluid imbalance, infection, scarring, and psychological sequelae following burn injury (6).

Current pediatric burn care typically involves wound assessment, cleansing, topical treatment, dressing changes, analgesic medication, infection prevention, scar management, and rehabilitation (7). Dressing changes are necessary for wound inspection, exudate removal, topical medication application, and prevention of contamination, yet they also represent one of the most painful and distressing procedures in burn care (8). Procedural pain during dressing changes often triggers fear and anxiety in pediatric burn patients, with pain, fear, and anxiety serving as core outcomes that directly influence children's tolerance of wound care (9, 10). Moreover, repeated exposure to painful procedures such as dressing changes can reduce cooperation, heighten anticipatory distress, and complicate rehabilitation, particularly when children associate dressing rooms or treatment equipment with prior painful experiences (11). Integrating pharmacological and non-pharmacological strategies can effectively manage pain in children with burn injuries, alleviating anxiety during medical procedures and ongoing care (12, 13).

Cognitive factors, such as attention and expectation, can profoundly shape pain perception and its modulation (14). Focusing on a wound or anticipating pain may amplify nociceptive input, whereas redirecting attention to competing stimuli can reduce perceived pain and distress (15, 16). Emerging evidence suggests that virtual reality (VR) interventions modulate sensory input and redirect attention from painful procedures through immersive visual and auditory stimulation (17). VR is a novel form of nonpharmacologic analgesia that can induce a greater improvement in positive emotions during medical procedures than traditional distraction methods (18). Prior studies have shown that VR intervention reduces pain unpleasantness, worst pain, and anxiety in pediatric patients undergoing needle-related procedures, with no reported side effects (19–21). An fMRI laboratory study comparing VR analgesia to opioid analgesia in healthy adults demonstrated that VR reduces subjective pain as effectively as a moderate dose of hydromorphone, and VR significantly decreases pain-related brain activity (22). Similarly, VR alone and opioids alone produced comparable reductions in pain-related brain activity (22). In pediatric burn care, VR can reduce dressing-related pain, fear, and anxiety, while recent evidence indicates that VR may improve children's emotional engagement during burn wound care (9, 23). Nevertheless, it remains unclear whether the benefits of VR are sustained across repeated dressing changes and whether VR can also improve observable behavioral distress, treatment cooperation, early rehabilitation participation, rescue analgesic requirement, and safety within the same clinical framework (9, 23, 24).

The present study aimed to examine the effects of a VR intervention on pediatric burn care during repeated dressing changes and early rehabilitation. Procedural pain, procedure-related fear, and behavioral distress were assessed across the first three dressing changes to capture responses to repeated wound care. Treatment cooperation, rehabilitation completion, dressing duration, rescue analgesic use, and adverse events were also recorded (7, 9, 23).

## 2 Methods

### 2.1 Study design and participants

This was a prospective, single-center, randomized controlled clinical study designed to evaluate the effect of immersive virtual reality (VR) as an adjunctive non-pharmacological intervention during burn dressing changes and early rehabilitation in pediatric patients. Children were recruited from the burn unit of No.910 Hospital of the Chinese People's Liberation Army Joint Logistic Support Force between January 2023 and March 2026. Eligible participants were randomly assigned to either the control group or the VR group.

Children aged 4–16 years with acute burn injuries requiring repeated dressing changes were considered eligible. The inclusion criteria were as follows: age between 4 and 16 years; diagnosis of thermal, scald, contact, or electrical burn; requirement for at least three dressing changes after enrollment; ability to understand age-appropriate pain and fear assessment tools; and written informed consent obtained from a parent or legal guardian. Assent was obtained from children when appropriate. The exclusion criteria were as follows: severe facial or head burns preventing safe use of a VR headset; severe visual, auditory, cognitive, or neurological impairment; history of epilepsy, vertigo, severe motion sickness, or intolerance to VR; unstable vital signs; requirement for deep sedation during dressing changes; severe infection or emergency surgery during the study period; and inability to complete pain or behavioral assessments.

The participants were randomly allocated to either the control group or the VR group. First, eligible children were assigned sequential identification numbers from 1 to 80 according to the order of enrollment. A random number was then generated for each identification number using SPSS version 26.0. Participants were ranked according to the size of the assigned random numbers. The 40 children with the largest random numbers were assigned to the control group, whereas the remaining 40 children were assigned to the VR group. The data collectors and researchers performing the statistical analyses were blinded to participant allocation.

### 2.2 Interventions

Children in both groups received standard burn care according to the institutional routine clinical protocol of the burn unit. Standard care included routine dressing changes (DC), pharmacological analgesia, psychological support, and early rehabilitation guidance, and was individualized according to wound condition and medical stability. Children in the VR group received the addition of immersive VR during dressing changes and early rehabilitation sessions. The VR intervention was delivered using a standalone head-mounted display, the PICO Neo 3 (Pico Interactive Inc., Beijing, China). With a 4 K resolution of 3,664 × 1,920 pixels, the device provides an immersive visual experience that helps children focus on audiovisual stimuli during painful procedures. The Chinese version of Tom and Jerry was used to achieve the virtual distraction activity. Before each dressing change, the VR headset was fitted and adjusted according to the child's head size and comfort. The VR animation began 10 min before dressing removal and continued throughout the procedure when tolerated. The same VR protocol was applied during the first three dressing changes after enrollment, designated DC1, DC2, and DC3. For early rehabilitation, VR was used during the first three sessions when clinically feasible, designated S1, S2, and S3. The child remained in a safe sitting or supine position during VR use, depending on the wound site and dressing requirements. If the child experienced dizziness, nausea, headache, eye discomfort, fear, refusal to continue, or if the headset interfered with wound care, the VR intervention was stopped immediately and the event was recorded.

### 2.3 Measurement and assessment

To evaluate the effect of VR distraction on pain relief during dressing changes, pain intensity was assessed immediately after each of the first three dressing changes using the Faces Pain Scale-Revised (FPS-R), scored from 0 to 10, with higher scores indicating greater pain intensity (25). In children with acute pain, a 2-point reduction or approximately 25% reduction on the FPS-R has been reported as a minimum clinically significant difference. Procedure-related fear was measured using the Children's Fear Scale (CFS), which consists of five facial expressions representing increasing levels of fear and is scored from 0 to 4, with higher scores indicating greater fear (26). Although the CFS has shown validity and reliability in assessing fear during painful medical procedures in children, a universally accepted minimum clinically important difference for pediatric burn dressing changes has yet to be established. Behavioral distress during dressing changes was assessed using the COMFORT Behavior Scale, an observer-rated instrument consisting of six behavioral domains scored from 1 to 5, with total scores ranging from 6 to 30 and higher scores indicating greater behavioral distress (27). Previous pediatric studies have shown that the COMFORT-B scale is responsive to treatment-related changes in pain or distress after analgesic or sedative interventions, although a setting-specific threshold for clinically meaningful improvement during pediatric burn dressing changes has not been established (28).

### 2.4 Statistical analysis

All statistical analyses were performed using SPSS version 26.0 (IBM Corp., Armonk, NY, USA). A two-sided *p*-value of less than 0.05 was considered statistically significant. Continuous variables were reported as mean ± standard deviation (SD), while categorical variables were presented as numbers and percentages. The normality of continuous variables and the homogeneity of variance across groups were assessed before analysis. For comparisons of continuous variables such as age, total burn surface area, FPS-R pain score, Children's Fear Scale score, COMFORT-B score, cooperation score, rehabilitation completion rate, and dressing change duration, the independent-samples *t*-test was applied. For categorical variables, including sex, burn depth, burn location, rescue analgesic requirement, and adverse events, the Chi-square test was used.

## 3 Results

### 3.1 Demographic characteristics of participants

Eighty eligible children were recruited for this study, with no significant differences in demographic or clinical characteristics observed between the two groups before intervention. The mean age in the control group was 10.25 ± 2.76 years, compared to 10.18 ± 2.60 years in the VR group, yielding no significant between-group difference (*p* = 0.9008). Sex distribution did not differ significantly between the VR and control groups (*p* = 0.6481). Regarding burn-related characteristics, there was no significant difference in TBSA, burn depth distribution, and burn location between the two groups (Table 1).

**Table 1:** Demographic characteristics of participants.

Characteristic	Control group (*n* = 40)	VR group (*n* = 40)	*p* value
Age, years	10.25 ± 2.76	10.18 ± 2.60	0.9008
Sex			0.6481
Male	23/40 (57.5)	25/40 (62.5)	
Female	17/40 (42.5)	15/40 (37.5)	
TBSA, %	7.88 ± 2.43	7.79 ± 3.19	0.8875
Burn depth			0.0674
Superficial partial-thickness	12/40 (30.0)	9/40 (22.5)	
Deep partial-thickness	14/40 (35.0)	24/40 (60.0)	
Mixed depth	14/40 (35.0)	7/40 (17.5)	
Burn location			0.1753
Upper limb	19/40 (47.5)	22/40 (55.0)	
Lower limb	12/40 (30.0)	6/40 (15.0)	
Trunk	1/40 (2.5)	5/40 (12.5)	
Multiple sites	8/40 (20.0)	7/40 (17.5)	

TBSA, total burn surface area; VR, virtual reality.

### 3.2 Changes in pain, fear, and behavioral distress during repeated dressing changes

During the first dressing change, no significant between-group differences were observed in procedural pain (FPS-R, *p* = 0.3597), procedure-related fear (Children's Fear Scale, *p* = 0.9018), or behavioral distress (COMFORT-B, *p* = 0.2348; Table 2). A similar trend was observed during the second dressing change, with no significant differences in FPS-R pain (*p* = 0.1142), Children's Fear Scale scores (*p* = 0.1710), or COMFORT-B scores (*p* = 0.0755; Table 2). By the third dressing change, the VR group showed lower scores than the control group for FPS-R pain (*p* = 0.0009), Children's Fear Scale score (*p* = 0.0116), and COMFORT-B score (*p* = 0.0048; Table 2).

**Table 2:** Procedural pain, procedure-related fear, and behavioral distress.

Measurement	Time	Control group (*n* = 40)	VR group (*n* = 40)	*p* value
FPS-R	DC1	6.10 ± 1.63	5.75 ± 1.77	0.3597
	DC2	6.20 ± 1.56	5.55 ± 2.05	0.1142
	DC3	6.10 ± 1.69	4.75 ± 1.79	0.0009
Children's Fear Scale	DC1	2.58 ± 0.81	2.55 ± 0.99	0.9018
	DC2	3.02 ± 0.62	2.80 ± 0.82	0.171
	DC3	2.65 ± 0.98	2.10 ± 0.93	0.0116
COMFORT-B Scale	DC1	21.88 ± 3.2	20.98 ± 3.50	0.2348
	DC2	21.85 ± 3.39	20.48 ± 3.44	0.0755
	DC3	21.57 ± 3.16	19.40 ± 3.53	0.0048

VR, virtual reality; DC, dressing change; FPS-R, faces pain scale-revised; COMFORT-B, COMFORT behavior.

**Figure 1:**
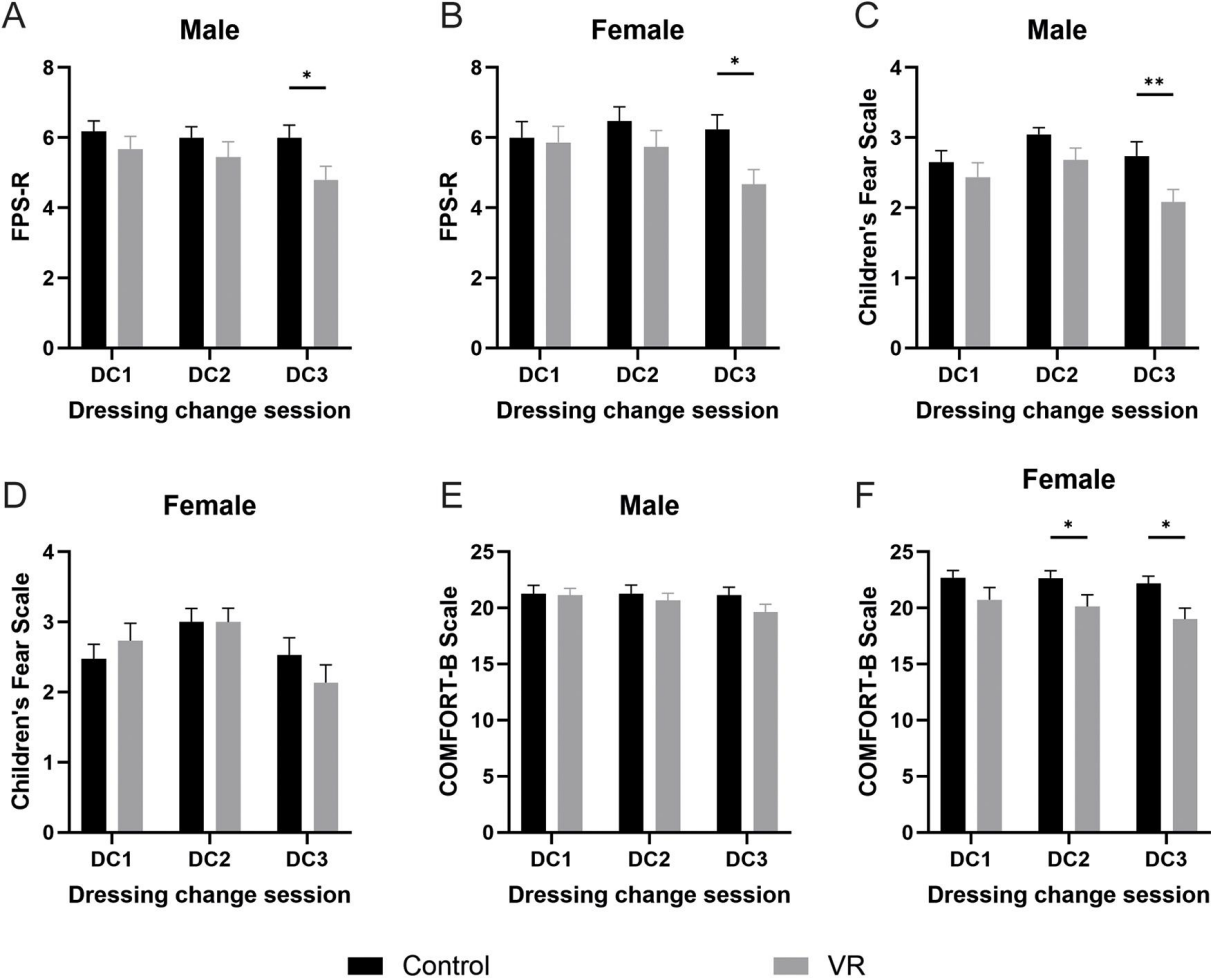
Sex-specific changes in procedural pain, procedure-related fear, and behavioral distress during repeated dressing changes. FPS-R **(A,B)**, Children's Fear Scale **(C,D)**, and COMFORT-B scores **(E,F)** are shown for male and female patients. Data are presented as mean ± SEM. *p < 0.05; **p < 0.01.

An exploratory sex-disaggregated analysis was conducted for procedural pain, procedure-related fear, and behavioral distress outcomes (Figure 1). Among male patients, FPS-R scores were comparable between the control and VR groups during DC1 and DC2, but lower FPS-R scores were observed in the VR group at DC3 (Figure 1A). For female patients, FPS-R scores showed no significant between-group differences at DC1 or DC2, although the VR group had lower scores at DC3 (Figure 1B). Regarding procedure-related fear, male patients in the VR group exhibited lower Children's Fear Scale scores at DC3 (Figure 1C), whereas no significant between-group difference was found among female patients across the three dressing changes (Figure 1D). For behavioral distress, COMFORT-B scores did not differ significantly between groups among male patients across any time point (Figure 1E). However, female patients in the VR group had lower COMFORT-B scores during DC2 and DC3 (Figure 1F).

### 3.3 Effects of VR on treatment cooperation and early rehabilitation participation

During the initial dressing change, no significant between-group difference was observed in dressing cooperation (*p* = 0.4457), and this difference remained nonsignificant during the second dressing change (*p* = 0.0952; Table 3). By the third dressing change, the VR group showed a higher dressing cooperation score than the control group (*p* = 0.0024; Table 3). For rehabilitation cooperation, no significant difference was found during the first rehabilitation session (*p* = 0.1483), whereas higher scores were found in the VR group during the second and third sessions (S2, *p* = 0.0352; S3, *p* = 0.0106; Table 3). The rehabilitation completion rate within 7 days was higher in the VR group, although the between-group difference did not reach statistical significance (*p* = 0.0627; Table 3). Similarly, no significant differences were observed between groups in dressing change duration across the three dressing changes (DC1, *p* = 0.3786; DC2, *p* = 0.5626; DC3, *p* = 0.3429; Table 3).

**Table 3:** Treatment cooperation and rehabilitation.

Measurement	Time	Control group (*n* = 40)	VR group (*n* = 40)	*p* value
Dressing cooperation score	DC1	3.08 ± 0.94	3.23 ± 0.80	0.4457
	DC2	3.25 ± 0.98	3.62 ± 1.00	0.0952
	DC3	3.05 ± 0.85	3.65 ± 0.86	0.0024
Rehabilitation cooperation score	S1	3.12 ± 0.65	3.38 ± 0.87	0.1483
	S2	3.23 ± 0.80	3.62 ± 0.87	0.0352
	S3	3.30 ± 0.76	3.77 ± 0.86	0.0106
Rehabilitation completion rate (%)	Within 7 days	72.63 ± 19.87	80.66 ± 18.16	0.0627
Dressing change duration (min)	DC1	25.40 ± 4.06	24.59 ± 4.17	0.3786
	DC2	24.37 ± 4.54	23.80 ± 4.30	0.5626
	DC3	24.45 ± 4.70	23.49 ± 4.29	0.3429

VR, virtual reality; DC, dressing change; S, rehabilitation session.

### 3.4 Rescue analgesic use and intervention safety

No significant between-group difference was observed in the overall requirement for rescue analgesia (*p* = 0.2561; Table 4). Similarly, the need for rescue analgesia did not differ significantly between groups during the first, second, or third dressing changes (DC1, *p* = 0.6331; DC2, *p* = 0.4459; DC3, *p* = 0.4123; Table 4). For safety, no significant between-group differences were found for specific adverse events, including dizziness (*p* = 0.1521), nausea (*p* = 0.3143), headache (*p* = 0.3143), and eye discomfort (*p* = 0.1521; Table 4).

**Table 4:** Rescue analgesic requirement and safety.

Feedback	Time	Control group (*n* = 40)	VR group (*n* = 40)	*p* value
Children requiring rescue analgesia (%)	Rescue analgesia	26/40 (65.0)	21/40 (52.5)	0.2561
	DC1	14/40 (35.0)	12/40 (30.0)	0.6331
	DC2	12/40 (30.0)	9/40 (22.5)	0.4459
	DC3	10/40 (25.0)	7/40 (17.5)	0.4123
Incidence of adverse events (%)	Dizziness	0/40 (0.0)	2/40 (5.0)	0.1521
	Nausea	0/40 (0.0)	1/40 (2.5)	0.3143
	Headache	0/40 (0.0)	1/40 (2.5)	0.3143
	Eye discomfort	0/40 (0.0)	2/40 (5.0)	0.1521

VR, virtual reality; DC, dressing change; S, rehabilitation session; NA, not applicable.

## 4 Discussion

This study evaluated immersive VR as an adjunctive intervention during repeated dressing changes and early rehabilitation in pediatric burn patients. The results revealed that VR did not significantly reduce procedural pain, procedure-related fear, or behavioral distress during the first two dressing changes, whereas significantly lower FPS-R, Children's Fear Scale, and COMFORT-B scores, together with higher dressing and rehabilitation cooperation scores, were observed in the VR group by the third dressing change, suggesting VR intervention can potentially improve clinical efficiency for dressing changes and early rehabilitation in a time-dependent manner.

Burn dressing changes expose children to multiple pain triggers, including dressing removal, wound cleansing, contact with inflamed tissue, and visual exposure to the wound, all of which may increase anticipatory fear and amplify pain perception. Pediatric procedural pain is not purely sensory; it is shaped by expectation, fear, prior painful memory, caregiver response, and the child's ability to shift attention away from the procedure (29). The reductions in pain, behavioral distress, and procedure-related fear observed in the VR group during repeated dressing changes were consistent with previous studies on VR-assisted pediatric burn dressing care (9). Earlier studies have also shown that immersive VR games can produce clinically meaningful pain relief during pediatric burn dressing procedures, with subjective pain scores decreasing by approximately 30%–50% compared with standard care (30, 31). According to the cognitive-affective model of pain, attentional redirection through immersive visual and auditory input is linked to reduced pain intensity, pain unpleasantness, and time spent thinking about pain in burn patients undergoing immersive VR (32). In our study, the use of familiar animation may have lessened the cognitive load needed to engage with VR, an important consideration for younger children who may struggle with complex interactive games during painful procedures. The use of smartphone-based virtual reality during pediatric burn dressing changes revealed lower self-reported pain and strong clinical feasibility, supporting the practicality of VR in real-world burn care (33). The analgesic and anxiolytic benefits of VR may not be observed immediately with a passive, cartoon-based program, instead, these effects become more apparent after repeated sessions, as children gradually adapt to the headset, audiovisual content, and dressing procedure. Similarly, a feasibility trial of home-based burn dressing care found that children and caregivers reported lower pain in the VR group during the fourth dressing change, suggesting that repeated VR exposure may enhance engagement and attenuate anticipatory distress over time (34). In addition, laboratory cold pressor studies in healthy children and young adults have reported greater analgesic effects with active VR than with passive VR games (35, 36). During a cold pressor experiment, VR was also shown to impose a stronger demand on cognitive resources in healthy children than the control condition, further supporting the role of attentional engagement in VR-mediated analgesia (37).

In this study, dressing cooperation improved significantly in the VR group by the third dressing change, and rehabilitation cooperation was higher during the second and third sessions, suggesting that VR may enhance patient adherence to both dressing changes and rehabilitation exercises. A pediatric desktop VR study on wound care reported lower FLACC pain scores, reduced anxiety, and greater joy during VR-assisted wound cleaning, indicating that VR can modulate both distress and affective engagement in burn care (23), VR intervention may help children remain still during dressing changes and participate more actively in early movement exercises by reducing fear and increasing procedural predictability. However, the higher rehabilitation completion rate observed in the VR group did not reach statistical significance, suggesting that improved cooperation during individual sessions may not be sufficient to produce a measurable increase in overall rehabilitation completion within 1 week. Rehabilitation completion is also influenced by wound site, pain fluctuations, dressing stability, sleep quality, family preferences, therapist scheduling, surgical plans, and medical restrictions (38); thus, a short-term audiovisual distraction intervention may be insufficient to overcome these multiple barriers. In contrast to the improvements in pain-related and cooperation-related outcomes, VR did not significantly reduce dressing change duration or the need for rescue analgesics. Dressing duration depends on wound size, anatomical location, exudate, dressing adherence, cleaning requirements, staff workflow, and clinical safety considerations, technical factors unlikely to change even when a child is calmer (39). A pilot randomized trial involving adult burn dressing changes highlighted practical challenges with VR implementation, including severe injury burden, opioid requirements, procedural interruptions, and difficulty maintaining VR engagement during complex wound care (40). Similarly, rescue analgesic use represents an indirect endpoint influenced by local medication protocols, clinician judgment, parental preference, baseline analgesic timing, and wound characteristics (41). The outpatient pediatric VR trial also emphasized the need for further research to determine whether VR provides opioid-sparing effects, despite its ability to reduce patient-reported pain (33).

In pediatric acute pain research, a 2-point reduction on the FPS-R has been linked to children reporting that their pain became “a little less,” whereas larger reductions have been associated with children reporting “much less” pain or no longer requiring additional analgesia (42, 43). The Children's Fear Scale has been validated as a brief measure of pain-related fear in children undergoing painful medical procedures; the existing validation literature supports its psychometric use rather than defining a burn dressing-specific minimal important difference (26). The COMFORT-B scale has shown responsiveness to treatment-related changes in pain or distress following analgesic and sedative interventions in critically ill children, though such evidence originates from a different clinical context than awake pediatric burn dressing procedures (28). Therefore, the psychometric improvements observed after repeated VR exposure may be best interpreted as reductions in procedural burden, while the degree of patient-perceived benefit remains inadequately captured by scale scores alone. Pediatric burn outcome research underscores the importance of patient- and parent-reported perspectives for capturing symptom severity, functional status, and overall well-being following treatment (44). Future studies should therefore integrate repeated quantitative assessments with child and caregiver global ratings, willingness to reuse VR, structured qualitative feedback, and responder analyses anchored to clinically meaningful thresholds.

The exploratory sex-stratified findings suggest that repeated VR exposure may differentially affect dimensions of procedural burden in male and female patients. In the male subgroup, reductions in self-reported pain and procedure-related fear emerged primarily in later sessions, supporting the role of VR as an attentional and affective distraction that may become more effective after repeated exposure rather than during the initial dressing session. Recent PAINDIFF recommendations emphasize routine consideration of sex and gender variables in pain research, as biological, developmental, psychological, and social factors may shape pain experience and treatment response (45). A recent pediatric burn VR trial further reported that VR engagement was associated with lower overall pain scores across age- and sex-based analyses, while female participants reported greater enjoyment during VR than male participants (46). In the present study, the female subgroup showed a broader behavioral response, with reduced observer-rated distress during later dressing sessions. Since the COMFORT-B scale captures observable behavioral distress rather than self-reported pain alone, the findings in the female subgroup may reflect changes in external distress expression, affective engagement, or behavioral regulation during repeated VR-assisted wound care (30). The delayed reduction in fear among male patients also aligns with the possibility that repeated exposure to a predictable audiovisual environment may reduce anticipatory fear during painful procedures; the Children's Fear Scale was developed to assess fear in pediatric procedural pain contexts (26).

This study has several limitations. As with other studies using nonpharmacologic interventions, blinding was not feasible, which may have influenced self-reported pain and observer-rated cooperation. The modest sample size also limited the statistical power for secondary outcomes, including rescue analgesia, adverse events, and rehabilitation completion. In addition, the VR content consisted of passive animation rather than interactive analgesic VR or rehabilitation-oriented VR, so the findings may not apply to highly immersive game-based systems. The outcomes were limited to early procedural responses, and longer-term endpoints such as dressing-related memory, scar discomfort, rehabilitation adherence after discharge, and functional recovery were not evaluated. VR effects may also vary by device type, content, immersion level, age, procedure type, and outcome scale; therefore, future multicenter studies should standardize VR protocols, compare passive and interactive content, and integrate objective behavioral or physiological measures with patient-reported outcomes. Age-related differences in VR engagement were not evaluated, suggesting that future studies could examine whether developmentally tailored VR content produces different effects in younger and older pediatric burn patients.

Future research should use adequately powered, multicenter randomized trials with prespecified subgroup analyses by age, burn depth, burn location, and procedure type. VR protocols should be reported in sufficient detail, including device type, level of immersion, content format, session timing, duration, number of sessions, criteria for discontinuation, and adverse-event monitoring. Future studies should also include standardized outcome sets, such as pain intensity, fear, behavioral distress, cooperation, rehabilitation adherence, rescue analgesic use, procedure duration, and longer-term functional recovery. To support guideline harmonization, future pediatric burn-care recommendations should clearly define the indications, contraindications, minimum safety requirements, outcome measures, and implementation procedures for VR-assisted procedural pain management and early rehabilitation.

## 5 Conclusion

In summary, the use of immersive VR during pediatric burn dressing changes was associated with lower pain, fear, and behavioral distress by the third dressing change and improved cooperation during later dressing and rehabilitation sessions. Exploratory sex-stratified analysis showed that VR-related improvements involved pain and fear among male patients and pain and behavioral distress among female patients. These findings suggest that VR may be a feasible and safe adjunctive strategy for repeated pediatric burn care procedures, with its greatest value likely lying in reducing procedural distress and improving children's willingness to participate in treatment.

## Data Availability

The raw data supporting the conclusions of this article will be made available by the authors, without undue reservation.
